# Hepatoprotective and Antiatherosclerotic Effects of Oleoylethanolamide-Based Dietary Supplement in Dietary-Induced Obesity in Mice

**DOI:** 10.3390/pathophysiology32020016

**Published:** 2025-04-18

**Authors:** Darya Ivashkevich, Arina Ponomarenko, Igor Manzhulo, Anastasia Egoraeva, Inessa Dyuizen

**Affiliations:** A.V. Zhirmunsky National Scientific Center of Marine Biology, Far Eastern Branch, Russian Academy of Sciences, Palchevskogo Str., 17, 690041 Vladivostok, Russia; owncean@yandex.ru (D.I.); arina.ponomarenko.93@mail.ru (A.P.); egoraeva.nastya@gmail.com (A.E.); duval@mail.ru (I.D.)

**Keywords:** oleoylethanolamide, OEA, MASLD, obesity, hypercholesterolemia

## Abstract

**Background:** Metabolic effects of oleoylethanolamide-based dietary supplement (OEA-DS) were studied in a model of dietary-induced obesity in mice. Obesity was induced by a 2-month high-fat, high-cholesterol diet, resulting in significant morphological changes in liver tissues and elevated cholesterol levels in the animals’ blood serum. Elevated levels of proinflammatory cytokines, oxidative stress, and hepatocyte apoptosis were also observed in the liver tissue. The aim of this study was to examine the mechanisms through which an OEA-based dietary supplement (OEA-DS) exerts a comprehensive influence on multiple aspects of the pathogenesis of MASLD, thereby demonstrating a robust hepatoprotective effect. **Methods:** mice were fed a high-fat, high-cholesterol diet with or without OEA-DS supplementation. Liver tissues and blood serum were analyzed for cholesterol levels, inflammatory markers (CD68, Iba-1, CD163, IL-1β, IL-6, TNFα), apoptotic markers (Bad, Bax, Bcl-2), nuclear receptors (PPAR-α, PPAR-γ, AdipoR1), and enzymes involved in lipolysis (Acox1, Cpt1a) and cholesterol metabolism (Ldlr, Furin, Pcsk9). Immunohistochemistry, Western blotting, and RT-PCR were used to assess protein expression and gene transcription. **Results:** administration of OEA-DS normalized cholesterol levels, decreased expression of inflammatory markers (CD68 and Iba-1), pro-apoptotic markers (Bad, Bax) and levels of pro-inflammatory cytokines (IL-1β, IL-6, TNFα). In parallel, the expression of nuclear receptors PPAR-α and PPAR-γ, adiponectin receptor 1 (AdipoR1), and anti-inflammatory (CD163) and anti-apoptotic (Bcl-2) markers have risen. OEA-DS administration induced the expression of liver lipolysis enzymes (Acox1, Cpt1a) and cholesterol metabolism factors (Ldlr, Furin), while simultaneously reducing the transcription of the proatherogenic factor Pcsk9. **Conclusions:** The results of this study suggest a complex action of OEA-DS in obesity-associated liver damage, which includes reduction of systemic inflammation.

## 1. Introduction

In recent decades, advances in healthcare and widespread lifestyle changes have contributed to a significant increase in diseases associated with affluence and overconsumption, particularly in developed nations. In particular, the epidemic of chronic liver disease is associated with the prevalence and continuous increase in the incidence of obesity worldwide [1]. Metabolic dysfunction-associated steatotic liver disease (MASLD) is a major cause of liver damage worldwide and affects a broad population across different age groups [2]. The presence of comorbidities in patients with MASLD results in an additional burden on health care systems. It is now known that MASLD often develops in combination with metabolic disorders characteristic of the metabolic syndrome. The relationship between non-alcoholic steatohepatitis (NASH) and obesity [3], hypertension [4], insulin resistance, and hypercholesterolemia [5] has been proven. Animal studies have repeatedly shown that diets high in fat, sugar, and cholesterol contribute to the development of acute liver injury and MASLD [6,7].

As obesity develops, in the case of peripheral obesity, neutral fat can be deposited as triacylglycerides (TAGs) either directly in subcutaneous adipose tissue [8] or affecting target organs, which is characteristic of visceral obesity. In visceral obesity, ectopic deposition of TAGs in hepatocytes underlies the development of MASLD. The basis of fat accumulation in the liver in MASLD is active fatty acid (FA) flux from adipose tissue and de novo lipogenesis against a background of increased glucose consumption and the development of insulin resistance [9]. Excessive influx of fatty acids into the liver, as well as disruption of glucose supply to cells, exceeds the enzymatic potential of hepatocytes, which leads to excessive accumulation of underoxidized products of fatty acid metabolism. As a consequence, lipotoxicity develops, which leads to the activation of lipid peroxidation and the development of oxidative stress. Against the background of these phenomena, the primary damage to hepatocytes, activation of immune cells, and development of high-grade inflammation occur, accompanied by fatty degeneration and apoptosis of liver cells [10]. Currently, specific therapy for MASLD is narrowly defined, and treatment is usually limited to dietary correction, increasing energy expenditure for weight loss, and the use of dietary supplements [11]. Accordingly, the search for new effective and safe pharmacological targets for the treatment of MASLD is a promising and socially important area. Pharmacological therapy should take into account various factors in the pathogenesis of MASLD and should be mainly aimed at reducing inflammation and normalizing lipid metabolism in the liver to reduce hepatocyte damage.

Peroxisome proliferator-activated receptors (PPAR) agonists are emerging as promising therapeutic options for MASLD treatment [12]. PPARs are known to act as regulators of transcription by stimulating or repressing the expression of numerous target genes [13]. It is important to note that PPAR controls genes that include key enzymes of lipid synthesis and breakdown, enzymes of glucose homeostasis, cholesterol metabolism, as well as genes involved in the development of obesity and inflammation [14]. Agonists of different PPAR subtypes are known to be well established in the therapy of metabolic disorders such as obesity [15], insulin resistance, and atherosclerosis [16], which are inextricably linked to the development of MASLD and often develop simultaneously. The PPAR-α receptor plays a central role in lipid metabolism in the liver, and its target genes are involved in mitochondrial FA oxidation, binding and activation, elongation, desaturation, triglyceride synthesis and cleavage, lipoprotein and apo-protein metabolism, gluconeogenesis, bile acid metabolism, and other related metabolic pathways [13]. Thus, this receptor plays a key role in the MASLD pathogenesis.

Oleoylethanolamide (OEA) is a member of the group of endogenous lipid mediators, N-acylethanolamides (NAE). OEA is predominantly characterized by marked agonism with the aforementioned PPAR-α receptor [17]. It is now well known that OEA is involved in various pathways regulating hunger and satiety as well as carbohydrate and lipid metabolism [18]. Pharmacological interest has mainly focused more on the anorexigenic effects of OEA, suggesting it as a promising candidate for the treatment of obesity and related metabolic disorders [19]. Its ability to suppress appetite and stimulate and prolong satiety has been repeatedly demonstrated in animal and human models of obesity [20]. In our previous work, we examined the pronounced PPAR-α-dependent anti-inflammatory activity of OEA in the development of diet-induced obesity, which is attributed to the modulation of immune cell activity [21].

The objective of this study was to examine the mechanisms through which an OEA-based dietary supplement (OEA-DS) exerts a comprehensive influence on multiple aspects of the pathogenesis of MASLD, thereby demonstrating a robust hepatoprotective effect.

## 2. Materials and Methods

### 2.1. Production of a Dietary Supplement

OEA-DS was produced from olive oil containing 72.3% oleic acid. The A.V. Zhirmunsky National Scientific Center of Marine Biology, Far Eastern Branch, Russian Academy of Sciences (NSCMB FEB RAS) served as the foundation for the dietary supplement’s production. The detailed methodology for obtaining the OEA-DS and the ratio of fatty acid ethanolamides in the resulting dietary supplement is described in detail in our previous work [21].

### 2.2. Experimental Model

Three-month-old female C57BL/6 mice were separated into four groups of 12 for the in vivo experiment. All animals were maintained under standard light/dark cycles (12 h of light and 12 h of darkness—LD 12:12). One group received standard feed, another (CTL+OEA-DS) received standard feed plus an OEA-DS supplement (200 mg/kg/day), a third received a modified feed to induce dietary obesity, and the last (DIO+OEA-DS) received the modified feed with the same OEA-DS supplement. The standard feed (Delta Feeds C-19, BioPro, Novosibirsk, Russia) consisted of cereals, high-protein components, vegetable oil, amino acids, organic acids, vitamins, and minerals (252 kcal/100 g). The modified feed (410 kcal/100 g) contained 60% standard feed, 20% sunflower oil, 20% sucrose, and 10% cholesterol.

The experiment was conducted for 2 months. Liver weight was measured at the end of the experiment. All animal procedures adhered to ethical standards and were approved by the relevant Russian Federation legal acts, the Basel Declaration, and the Biomedical Ethics Commission of the NSCMB FEB RAS (protocol № 16150-208-01/122 of 31 January 2023).

### 2.3. Immunohistochemical Staining

For histological and immunohistochemical analyses, mice were anesthetized using 3% isoflurane in 100% oxygen delivered via a rodent anesthesia vaporizer (VetFloTM, Torrington, CT, USA). After inhibition of their primary reflexes, animals were transcardially perfused with ice-cold solutions of 10% formalin and phosphate buffer. The liver tissues were then extracted and stored in 10% formalin for a 24 h period at 4 °C. Subsequent to phosphate buffer washing, xylene preparation and direct paraffin embedding were performed.

To suppress endogenous peroxidase, paraffin sections of liver (7 μm thick) were deparaffinized and then treated for 15 min in 3% hydrogen peroxide solution. They were then washed three times for 10 min with 0.1 M phosphate buffer (pH 7.2). Then, blocking buffer consisting of 0.1 M phosphate buffer (pH 7.2) with 2% bovine serum albumin (SC-2323, Santa Cruz Biotechnology, Santa Cruz, CA, USA), 0.1% Tween20 AppliChem, Darmstadt, Germany), and 0.25% Triton X-100 (Gerbu, San Diego, CA, USA) was applied to the slides and left for incubation for one h. Thereafter, primary antibodies, previously dissolved in the blocking buffer, were added to the markers. The primary antibodies utilized in this study included: PPAR-α (1:1000, ab245119, Abcam, Cambridge, MA, USA); Bcl-2 (1:1000, ab59 348, Abcam, UK); AdipoR1 (1:1000, ab70362, Abcam, Waltham, UK); and Iba-1 (1:2000, ab108539, Abcam, Waltham, UK). Primary antibody incubation was carried out throughout the entire night at 4 °C. Concurrently, negative control incubation was conducted, which did not include the main antibodies. Subsequently, the slides were incubated with a secondary antibody solution (Biotinylated Goat Anti-rabbit IgG (H+L), ab178846, Abcam, Waltham, UK) for a period of 15 min. This was followed by three additional 10 min washes using phosphate buffer (0.1 M, pH 7.2). The slides were then incubated in streptavidin solution (ab64269, Abcam, UK) for 10 min and in chromogen solution (Nova Red, Vector Laboratories, Burlingame, CA, USA) for 5 min. After stopping the staining reaction with distilled water, the slides were dehydrated and then coated with plating medium.

The area of immunohistochemical staining of every fifth serial section on the preparations was assessed using the ImageJ 1.41 software package (NIH, Bethesda, MD, USA). For each marker, the percentage ratio of the area of the immunopositive region to the total area was calculated.

### 2.4. Histological Staining

Subsequent to the dewaxing process, liver slices were immersed in Mayer’s hematoxylin solution (BioVitrum, Saint Petersburg, Russia) for a period of two minutes. Thereafter, the slices were rinsed for a duration of fifteen minutes under running water. Subsequently, the slices were stained with eosin (BioVitrum, Saint Petersburg, Russia) for histological staining. Following a five-second immersion in a concentrated eosin solution, the slides were rinsed three times in varying amounts of 96% ethanol. Thereafter, the slices were wrapped in mounting media and placed in xylene for a period of ten minutes.

### 2.5. Measurement of Serum Cholesterol Levels

To assess cholesterol levels, all animals were deprived of food the night before the biomaterial was taken. Blood obtained from the right atrium was measured three times using EasyToch test strips (Bioptik, New Taipei City, Taiwan). The mean of the three measurements was calculated for each animal, and then the differences between the groups were evaluated directly.

### 2.6. Cell Culture

In vitro investigations were conducted using the HepG2 hepatocarcinoma cell line. The cells were cultivated in standard DMEM media supplemented with 10% FBS, 1% penicillin/streptomycin, and essential amino acids. The cultivation took place at 37 °C in a humidified environment with 5% CO_2_. At least four independent runs of each in vitro experiment were conducted. For Western blotting, PCR analysis, and MDA production investigations, cells were seeded onto T-25 culture vials (1 × 10^5^ cells/cm^2^). For investigations of cell toxicity, cells were seeded into 96-well microplates (1 × 10^5^ cells/cm^2^).

### 2.7. Cytotoxicity Assay

The MTS test was used to evaluate the cytotoxic activity of OEA-DS on HepG2 cell culture. Following seeding of the cells (1 × 10^5^ cells/cm^2^) in clear 96-well plates, the cells were incubated for 1 h in standard media. Following the adhesion of the cells, the medium was supplemented with OEA-DS at concentrations of 0.01, 0.1, 1, and 10 µg/mL. The cells were then incubated for 24 h in enriched medium. The negative control consisted of cells cultivated on an additive-free medium. The following day, each well was filled with MTS reagent (ab197010, Abcam, UK) and incubated for 2 h at 37 °C. At a wavelength of 490 nm, optical density was measured using a microplate reader (Biorad, USA). Percentages of the results in relation to the negative control were displayed.

### 2.8. Oxidative Stress Assessment

Malone dialdehyde (MDA) levels in the liver tissues with diet-induced obesity and in LPS-induced HepG2 cell culture were assessed using the Lipid Peroxidation Assay Kit (MAK085, Sigma, St. Louis, MO, USA). T-25 culture vials were used for cell seeding (1 × 105 cells/cm^2^). After 1 h of cell adhesion, the culture medium was replaced with medium supplemented with OEA-DS at concentrations of 0.01, 0.1, 1, and 10 µg/mL. LPS (1 mg/mL) was then added to activate the cells. Cells activated with LPS alone served as the positive control, and cells in standard culture medium served as the negative control. After 24 h, cells were passaged with Versene/EDTA solution (HIMedia, Maharashtra, India) and stored at −80 °C until further use. Liver tissues were homogenized using a pestle, HepG2 cells were homogenized using an ultra-sonic homogenizer, and all further manipulations were performed according to the manufacturer’s instructions. Optical density was assessed using a microplate reader (Biorad, USA) at a wavelength of 532 nm. The results for liver tissues were presented in relation to the “CTL” group, for cell culture, in relation to the negative control without LPS addition.

### 2.9. Western Blotting

HepG2 hepatocarcinoma culture was seeded onto T-25 vials and treated with the OEA-DS at concentrations of 0.1, 1, and 10 µg/mL. Subsequently, the cells were ultrasonically homogenized in phosphate buffer (7.2 pH) augmented with 150 mM serine protease inhibitor (PMSF, Helicon GC207002, Moscow, Russia) following a 24 h incubation period. Liver tissues were manually ground with a pestle, and phosphate buffer mixed with 150 mM PMSF was employed to homogenize the tissues. The serum was simply diluted with 150 mM PMSF in phosphate buffer, without undergoing homogenization.

The samples’ protein concentrations were adjusted to 2 mg/mL. Subsequently, loading buffer (2× Sample Buffer, Biorad, Hercules, CA, USA) supplemented with 5% 2-mercaptoethanol (Sigma-Aldrich, M6250, St. Louis, MO, USA) was added to the samples in equal amounts. Subsequently, the samples were subjected to an additional five-minute incubation at 94 °C in a water bath. For the electrophoresis analysis, Protean mini gel Any kDa (Biorad, USA) ready-to-use gel cartridges and a Spectra Multicolor Broad Range Protein Ladder (Thermo Fisher, Waltham, MA, USA) molecular ladder were employed in a Biorad chamber. The examination of the analyzed markers in the liver and loading control utilized the same single well loading of 40 mg/well. Subsequently, the Transblot Turbo Transfer kit and Transblot Turbo system (Biorad, Hercules, CA, USA) were employed to transfer the samples to PVDF membranes. The membranes were then incubated for an hour in blocking buffer (0.1 M phosphate buffer, pH 7.2, with 2% bovine serum albumin) following the transfer process. The membranes were subsequently stored at 4 °C for a period of 12 h in solutions of primary antibodies targeting the following markers. Prior to this, the membranes were subjected to three cycles of cleaning, each lasting 5 min, using a phosphate buffer that was supplemented with 0.1% Tween 20 (PBS-T). The primary antibodies utilized included α-Tubulin (1:1000, ab7291, Abcam, Cambridge, MA, USA), PPAR-α (1:500, ab45036, Abcam, Cambridge, MA, USA), and ASAHL (1:1000, sc-100470, Santa Cruz Biotechnology, Santa Cruz, CA, USA), PPAR-γ (1:500, ab45036, Abcam, Cambridge, MA, USA), IL-1β (1:1500, ab9722, Abcam, Cambridge, MA, USA), IL-6 (1:1500, ab208113, Abcam, Cambridge, MA, USA), TNFα (1:1000, Thermo Fisher 701135, Thermo Fisher Scientific, Waltham, MA, USA), Bax (1:2000, ab325 03, Abcam, Cambridge, UK), Bcl-2 (1:2000, ab59348, Abcam, Cambridge, UK), and AdipoR1 (1:2000, ab70362, Abcam, Cambridge, UK) dissolved in a phosphate-buffered saline (PBS) buffer containing 0.02% bovine serum albumin (BSA) and 0.1% Tween 20.

Loading of each marker, including the control α-tubulin, on a separate gel was performed in connection with a modified protocol used for Western blotting analysis of a large set of markers in tissue samples from experimental animals, many of which have similar molecular weights and cannot be detected on a single membrane. The following day, the membranes were incubated for 1 h with secondary anti-mouse (Vector Laboratories, PI-2000, Burlingame, CA, USA) and anti-rabbit (Vector Laboratories, PI-1000, USA) antibodies dissolved in PBS-T at a concentration of 1:2000. This was followed by 3–5 min long washes with PBS-T solution. The chemiluminescence reaction was catalyzed by Western Blot ECL Substrate (Biorad, USA), and the detection process was carried out using a ChemiDoc gel documentation system (Biorad, USA). Following a five-minute incubation period with one milliliter of substrate, the membrane was detected. A single marker was detected on each membrane, in accordance with the test parameters that had been previously described. The software program ImageJ 1.41 (NIH, Bethesda, MD, USA) was used to analyze the acquired images. Images of the bands are provided in Supplementary Materials S1; full images of the gels are provided in Supplementary Materials S2.

### 2.10. RT-PCR Assay

Real-time PCR (rt-PCR) was utilized to assess the expression levels of the genes Acox1, Spt1a, Pcsk9, Ldlr, and Furin. An aerodent anesthetic vaporizer (Vet Flo TM, Kent Scientific Corporation, Torrington, CT, USA) was employed to sedate four animals per group using isoflurane. Liver tissues were promptly extracted and preserved in IntactRNA (Evrogen, Moscow, Russia). The fixed material was stored in refrigerators at +4 °C for a 24-h period. Primer Premier 5 software (Premier Biosoft International, Palo Alto, CA, USA) was utilized to create primers for rt-PCR based on sequences from the NCBI database. Evrogen, a company based in Moscow, Russia, was responsible for creating the primers. The “Aurum Total RNA Fatty and Fibrous Tissue” kit (Bio-Rad, Watford, UK) was employed to isolate total RNA, and agarose gel electrophoresis and a spectrophotometer (Thermo Fisher, Waltham, MA, USA) were utilized to determine its concentration. To create the first strand of cDNA, the ProtoScript^®^ II kit (New England Biolabs, Hitchin, UK) was employed. The RT-PCR was carried out on a CFX96 amplifier (Bio-Rad, Hercules, CA, USA) and a SYBER Green I RT-PCR kit (Evrogen, Moscow, Russia) in a total volume of 20 μL, comprising 1 μL of cDNA, 0.5 μM primer, and nuclease-free water. For every primer pair, the same mixture lacking cDNA served as a control. Glyceraldehyde-3-phosphate dehydrogenase (GAPDH) and beta-actin (Actb) were utilized as comparative genes. Microsoft Excel 2010 and Bio-Rad CFX Manager 2.1 were employed for the processing of the acquired data. An intact animal group was designated as the control sample. All target gene expression values were normalized to the reference genes Actb, GAPDH. The relative expression levels were analyzed by the 2-ΔΔCt method.

### 2.11. Statistical Analysis

The statistical analysis and graphical interpretation were carried out using GraphPad Prism 8.00 (GraphPad Software, San Diego, CA, USA). The animal material was subjected to Tukey’s multiple comparisons analysis following a two-way ANOVA. Subsequently, Tukey’s multiple comparisons analysis was performed after one-way ANOVA, and the Student’s *t*-test was utilized to analyze the cell culture data. Statistical significance was considered as *p* < 0.05. The data were presented as mean ± SEM.

## 3. Results

### 3.1. OEA-DS Administration Alleviates Hepatic Steatosis

**Figure 1:**
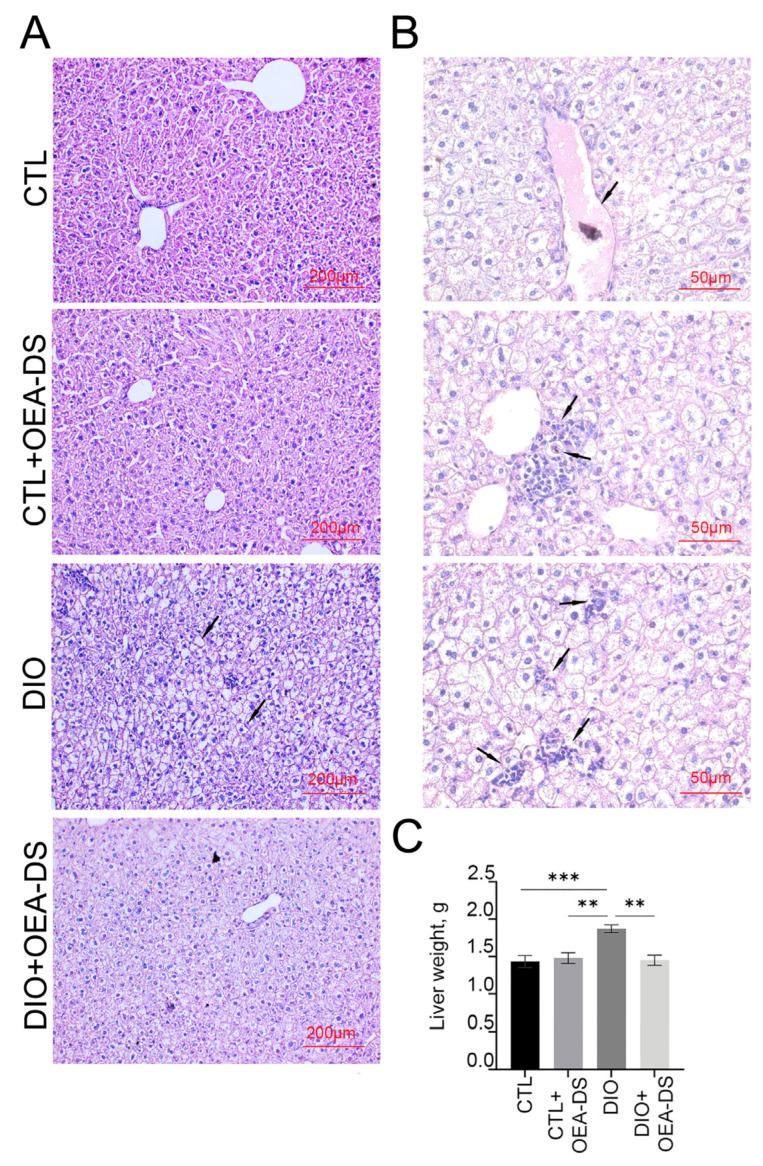
Morphological analysis of liver tissues (**A**). The arrows in the figure indicate the main pathological phenomena observed in DIO modeling (**B**): blood vessel stasis, immune cell accumulation in the central vasculature, damaged hepatocytes with fat vacuoles, foci of hepatocytes subjected to fat necrosis, apoptotic cells, and immune cell accumulation around damaged and destroyed hepatocytes. Liver weight estimation (**C**). Data are presented as mean ± SEM, ** *p* < 0.01, *** *p* < 0.001, (Two-way ANOVA followed by Tukey’s multiple comparisons analysis). The number of animals in the group = 6; the period of OEA-DS administration was 2 months; the dose of OEA-DS was 200 mg/kg per day.

On histological liver preparations in the “DIO” group revealed a characteristic sign of intensive fatty dystrophy development, which is much less pronounced in the “DIO+OEA-DS” group (Figure 1A). Fine-grained fatty degeneration affected most hepatocytes in the DIO group; groups of cells contained large lipid droplets included in the cytoplasm (Figure 1A,B). At the sites of necrotic hepatocytes, areas of fatty-protein detritus formed, fat droplets merge and form extracellular clusters around which there is a cellular reaction. Lymphoid infiltration appeared in the central parts of the lobules around the vessels. Sinusoid spaces and portal tracts were dilated, and stasis and hepatic vascular hemorrhage were present. Both unchanged nuclei and those with different degrees of damage were present; vacuolization of nuclei, pyknosis, and, in some cells, nuclei were absent altogether. In the foci of RES (Reticulo-endothelial system) cells accumulating around the damaged hepatocytes, apoptotic cells are present (Figure 1B). In the sections of the “CTL+OEA-DS” group, the morphological features of a normal intact liver are observed; there are no significant morphological differences from the “CTL” group (Figure 1A). The development of hepatic steatosis induced by the hypercaloric diet was reflected in changes in liver weight. In the “DIO” group, intensive fat deposition in the liver was accompanied by an increase in its weight (1.9 ± 0.2 g), 35% higher than the liver weight of control animals (1.4 ± 0.2 g). OEA-DS treatment in obese animals attenuated liver mass gain, resulting in a liver weight comparable to that of animals receiving a standard diet (1.5 ± 0.1 g). The addition of OEA-DS to the standard diet did not affect liver weight; the values of the “CTL+OEA-DS” group were almost equivalent to the control (1.5 ± 0.2 g). Two-way ANOVA revealed significant effects of obesity (F (1, 20) = 9.025; *p* = 0.0070), OEA-DS administration (F (1, 20) = 7.611; *p* = 0.0121), and their interaction (F (1, 20) = 11.87; *p* = 0.0026) on liver weight. Thus, the development of fatty liver dystrophy in DIO favors an intensive increase in liver weight, while the use of OEA-DS contributes to the inhibition of these processes (Figure 1C).

### 3.2. OEA-DS Administration Causes Activation of PPAR-α and PPAR-γ Receptors and Adiponectin Receptor-1 (AdipoR1) in Liver and Serum

**Figure 2:**
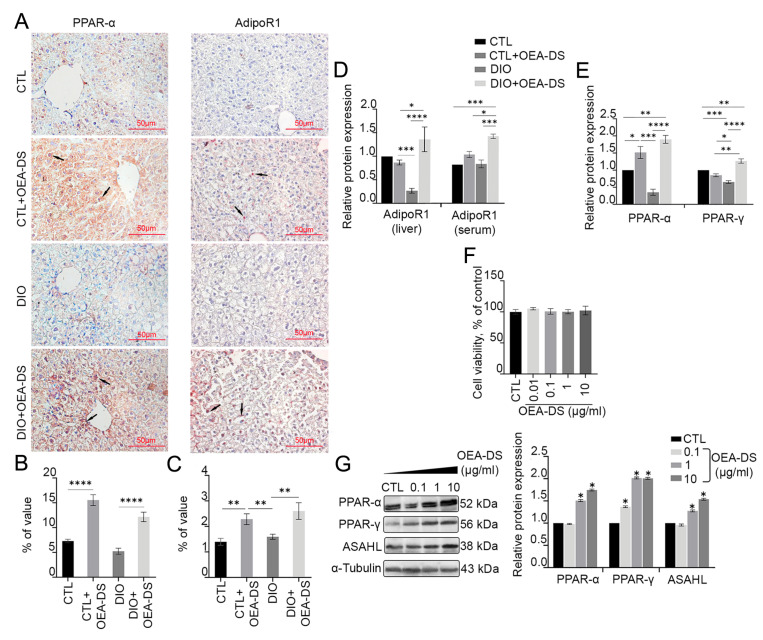
Immunoperoxidase reaction in liver tissue for PPAR-α and AdipoR1 marker (**A**). Arrows in the photos indicate immunopositive cells for each marker. PPAR-α is mainly expressed in hepatocytes, with low expression levels in non-parenchymal cells, AdipoR1 expressed on hepatocytes, hepatic stellate cells, and Kupffer cells. The graph estimates the percentage of immunopositive staining area (**B**,**C**). Western blot analysis of AdipoR1 expression in liver tissue and serum (**D**) and PPAR-α and PPAR-y receptors in liver (**E**), expression levels normalized to alpha-tubulin and expressed relative to control. Data are presented as mean ± SEM, ** *p* < 0.01, *** *p* < 0.001, **** *p* < 0.0001 (Two-way ANOVA followed by Tukey’s multiple comparisons analysis). Evaluation of OEA-DS cytotoxicity by MTS method (0.1, 1, 10 µg/mL) (**F**). Western blot analysis of PPAR-α, PPAR- γ, and ASAHL levels in HepG2 hepatocarcinoma lysates (**G**). Expression levels normalized to alpha-tubulin and expressed relative to control are plotted. Data are presented as mean ± SEM, * *p* < 0.05 (Student’s *t*-test). The number of animals in the group = 6 (for IHC) and 3 (for WB); the period of OEA-DS administration was 2 months; the dose of OEA-DS was 200 mg/kg per day. Number of in vitro samples = 8 (replicates), each experiment was performed independently at least 3 times.

Immunohistochemical assessment of PPAR-α receptor distribution area in liver tissue revealed significant effects of obesity (F (1, 46) = 13.17; *p* = 0.0007) and OEA-DS administration (F (1, 46) = 102.1; *p* < 0.0001) separately, but not their interaction, as determined by two-way ANOVA. Thus, the development of DIO and OEA-DS administration both influenced PPAR-α receptor expression in the liver. Multiple comparisons analysis showed that OEA-DS administration to animals receiving a standard diet (7.3 ± 0.4% in the “CTL” group; 15.5 ± 1.6% in the “CTL+OEA-DS” group) and animals with DIO (5.2 ± 0.6% in the “DIO” group; 12.2 ± 0.9% in the “DIO+OEA-DS” group) increased PPAR-α expression levels by more than 100% (Figure 2B).

Two-way analysis of variance evaluating the AdipoR1-immunopositive region in the liver revealed significance only for OEA-DS administration (F (1, 42) *p* = 18.89; *p* < 0.0001). Multiple comparisons analysis also demonstrated that adding OEA-DS to animals with a standard diet (1.4 ± 0.1% in the “CTL” group; 2.3 ± 0.2% in the “CTL+OEA-DS” group) and obese animals (1.6 ± 0.1% in the “DIO” group; 2.6 ± 0.3% in the “DIO+OEA-DS” group) in-creased AdipoR1 levels by more than 60%. Consequently, AdipoR1 expression depended solely on OEA-DS administration (Figure 2C).

Western blot analysis of liver tissue and serum further revealed the effect of OEA-DS on protein synthesis of PPAR-α and AdipoR1 receptors (Figure 2D,E). OEA-DS administration increased PPAR-α receptor expression in both animals with a standard diet (52% above the control level) and obese animals (90% above the control level). Furthermore, OEA-DS administration to obese animals increased AdipoR1 expression in both liver and serum (40% above control levels in liver, 59% above control levels in serum). Meanwhile, OEA-DS administration to animals with a standard diet promoted an increase in Adi-poR1 levels only in serum (19% above control levels), but not in the liver. At the same time, the development of DIO significantly decreased both PPAR-α (65% below the control level) and AdipoR1 (70% below the control level) in the liver. When assessing PPAR-α synthesis, two-way ANOVA revealed significant effects of OEA-DS administration (F (1, 8) = 92.17; *p* < 0.0001) as well as factor interactions (F (1, 8) = 24.06; *p* = 0.0012). When analyzing Adi-poR1 expression in the liver, two-way ANOVA revealed significance of OEA-DS insertion (F (1, 8) = 7.640; *p* = 0.0245), as well as factor interactions (F (1, 8) = 12.25; *p* = 0.0081), while serum ANOVA revealed the significance of obesity factors (F (1, 8) = 7.895; *p* = 0.0229) and OEA-DS administration (F (1, 8) = 17.50; *p* = 0.0031). Thus, OEA-DS directly stimulates the synthesis of these receptors in both healthy livers and livers affected by steatosis.

Additionally, treatment of the hepatocarcinoma cell line with OEA-DS at a concentration of 1 µg/mL and higher significantly increased the expression of PPAR-α and PPAR-γ receptors, as shown by Western blotting (when cells were treated with OEA-DS at a concentration of 10μM, PPAR-α increased by 74% compared to the control, and PPAR-γ by 104% compared to the control). Furthermore, the synthesis of the NAEs metabolic enzyme ASAHL increased by 54% compared to the control at the maximum concentration of OEA-DS (Figure 2G). At the same time, none of the studied concentrations of OEA-DS exhibited a cytotoxic effect on cells (Figure 2F).

### 3.3. Administration of OEA-DS Leads to a Decrease in Inflammatory Reactions in the Liver and Oxidative Stress

**Figure 3:**
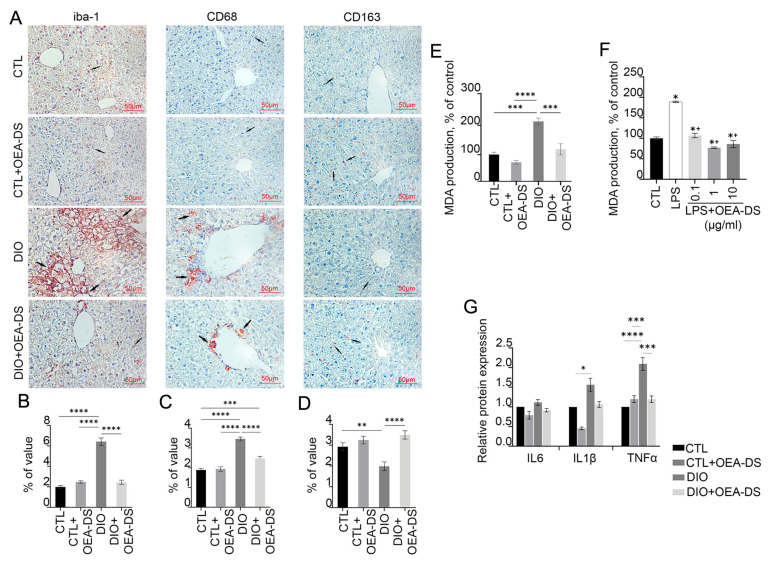
Immunoperoxidase reaction for markers Iba-1, CD68, and CD163 in liver tissue (**A**). Arrows in the photos indicate immunopositive cells for each marker. These markers have been found to stain monocyte-macrophage lineage cells. Iba-1 is characteristic of the total pool of macrophages, CD68 is characteristic of the pro-inflammatory phenotype of macrophages (M1), and CD163 is characteristic of the anti-inflammatory phenotype of macrophages (M2). The graphs estimate the percentage of immunopositive staining area (**B**–**D**). Analysis of MDA production in liver tissues (**E**) and in HepG2 cell culture (**F**). Data are mean ± SEM, n = 8 (number of samples analyzed), * *p <* 0.05 compared with “CTL”, + *p* < 0.05 compared with “LPS” (Student’s *t*-test). Western blot analysis of pro-inflammatory cytokine expression in liver tissue (**G**), expression levels normalized by alpha-tubulin and expressed relative to control. Data are presented as mean ± SEM, * *p* < 0.05, ** *p* < 0.01, *** *p* < 0.001, **** *p* < 0.0001 (Two-way ANOVA followed by Tukey’s multiple comparisons analysis). The number of animals in the group = 6 (for IHC) and 3 (for WB); the period of OEA-DS administration was 2 months; the dose of OEA-DS was 200 mg/kg per day. Number of in vitro samples = 8 (replicates), each experiment was performed independently at least 3 times.

DIO-induced obesity resulted in a significant increase in the density and formation of focal Iba-1-positive macrophage clusters in the liver, particularly in the central regions of hepatic lobules. In the “DIO” group, macrophages occupied a percentage of area (6.4 ± 0.4%) more than 2.5 times higher than in the control group (2.0 ± 0.1%) and the “CTL+OEA-DS” group (2.5 ± 0.1%). OEA-DS administration to obese animals promoted a decrease in the activity of Iba-1-positive cells (2.4 ± 0.2%), bringing it almost to control levels (Figure 3A). When assessing the area of distribution of Iba-1-positive macrophages, two-way ANOVA revealed a significant effect of obesity (F (1, 75) = 83.05; *p* < 0.0001), OEA-DS administration (F (1, 75) = 52.75; *p* < 0.0001), and their interaction (F (1, 75) = 83.91; *p* < 0.0001) (Figure 3B).

Quantitative assessment showed a significant increase in the proportion of CD68-positive cells during the development of DIO. Cell clusters predominantly resided in the central regions of the lobules. The “DIO” group showed a significant increase (3.5 ± 0.1%), more than 50%, compared to the “CTL” (1.9 ± 0.1%) and “CTL+OEA-DS” (1.9 ± 0.1%) groups. In the “DIO+OEA-DS” group, activation of CD68-positive cells was significantly less, 25% higher than the control group (2.5 ± 0.1%) (Figure 3A). For CD68, two-way ANOVA revealed significant effects of obesity (F (1, 76) = 108.4; *p* < 0.0001), OEA-DS application (F (1, 76) = 21.68; *p* < 0.0001), and a significant interaction effect of these factors (F (1, 76) = 26.07; *p* < 0.0001) (Figure 3C).

Evaluation of CD163 marker distribution in liver parenchyma showed that DIO development caused a 47% decrease in marker expression compared to controls (2.9 ± 0.2% in the “CTL” group; 2.0 ± 0.2% in the “DIO” group), while OEA-DS administration to animals receiving a standard diet caused no significant difference (3.2 ± 0.2%), but treatment of obese animals resulted in a 20% increase in marker levels compared with controls (3.5 ± 0.2%) (Figure 3A). In the case of CD163, two-way ANOVA revealed a significant effect of OEA-DS administration (F (1, 56) = 21.40; *p* < 0.0001) and interaction of factors (F (1, 56) = 9.009; *p* = 0.0040), with no significant effect of obesity (Figure 3D).

Taken together, the results indicate that obesity provokes macrophage activation in the liver. OEA-DS administration reduces the activity of pro-inflammatory cellular elements to control levels, while increasing the fraction of anti-inflammatory macrophages in the liver. Analyzing the levels of pro-inflammatory cytokine synthesis in the liver by Western blotting (Figure 3G), in the case of IL-1β, two-way ANOVA analysis revealed significant effects of obesity (F (1, 8) = 8.935; *p* = 0.0174) and OEA-DS administration (F (1, 8) = 7.157; *p* = 0.0281), but not their interactions. For TNFα, two-way ANOVA revealed significant effects of obesity (F (1, 8) = 14.87; *p* = 0.0048), OEA-DS administration (F (1, 8) = 6.188; *p* = 0.0377), and the interaction of these factors (F (1, 8) = 15.03; *p* = 0.0047). In contrast, in the case of IL-6, only a significant effect of OEA-DS administration was found (F (1, 8) = 6.912; *p* = 0.0302).

DIO increased the levels of pro-inflammatory cytokines IL-1β and TNFα in the liver. However, OEA-DS administration to animals with DIO reduced the expression of these cytokines, with no significant effect observed in animals receiving a standard diet. Furthermore, the rate of fatty dystrophy development correlated directly with the intensity of lipid peroxidation activation processes in the liver. This was reflected in a significant increase in the MDA level in the “DIO” group, which was more than 2 times higher compared to the control group. OEA-DS therapy reduced MDA levels in both obese animals and animals on a standard diet. Two-way ANOVA revealed significant effects of obesity (F (1, 12) = 41.81; *p* < 0.0001), OEA-DS administration (F (1, 12) = 24.99; *p* = 0.0003), and their interaction (F (1, 12) = 7.896; *p* = 0.0157), indicating that OEA-DS possesses pronounced antioxidant activity in both standard diet and DIO conditions (Figure 3E).

LPS-induced inflammation also increased MDA production in hepatocarcinoma cells (94% higher than the control level). Pretreatment of cells with OEA-DS at concentrations (0.1–10 μg/mL) significantly decreased MDA production (*p* < 0.05) (Figure 3F).

### 3.4. OEA-DS Reduces the Intensity of Apoptotic Processes in the Liver

**Figure 4:**
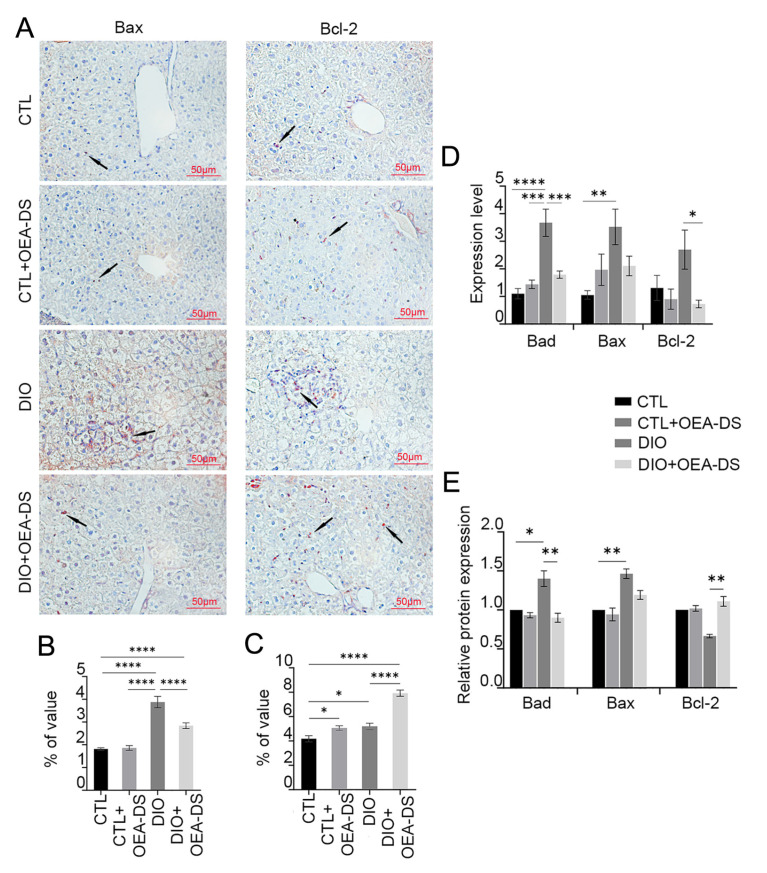
Immunoperoxidase reaction for Bax and Bcl-2 markers in liver tissue (**A**). Arrows in the photos indicate immunopositive cells for each marker. Bax is a pro-apoptotic marker, Bcl-2 is an anti-apoptotic marker. Both markers are mainly expressed in hepatocytes. The graphs estimate the percentage of immunopositive staining area (**B**,**C**). Western blot analysis of liver tissues for Bad, Bax, and Bcl-2 factors (**E**). Expression levels normalized to alpha-tubulin and expressed relative to control are plotted. PCR analysis of Bad, Bax, and Bcl-2 gene expression levels in liver tissue (**D**). Data are presented as mean ± SEM, * *p* < 0.05, ** *p* < 0.01, *** *p* < 0.001, **** *p* < 0.0001 (Two-way ANOVA followed by Tukey’s multiple comparisons analysis). The number of animals in the group = 6 (for IHC), 3 (for WB), and 3 (for rt-PCR); the period of OEA-DS administration was 2 months; the dose of OEA-DS was 200 mg/kg per day.

Fatty liver dystrophy development activated apoptotic processes, increasing the number of Bax-positive cells by more than 2 times compared to the control (1.8 ± 0.1% in the “CTL” group; 3.9 ± 0.3% in the “DIO” group). OEA-DS addition to animals with a standard diet had no significant effect on the expression of pro-apoptotic markers (1.9 ± 0.1%). However, in obese animals, OEA-DS addition led to a 40% decrease in the proportion of Bax-positive cells compared to the “DIO” group (2.8 ± 0.1%) (Figure 4A). Two-way ANOVA, when analyzing the distribution of Bax-positive cells in the liver, revealed significant effects of obesity (F (1, 74) = 93.23; *p* < 0.0001), OEA-DS administration (F (1, 74) = 9.901; *p* = 0.0024), and the interaction of these factors (F (1, 74) = 11.80; *p* = 0.0010) (Figure 4B).

The DIO diet resulted in a 20% increase in the area of Bcl-2 distribution (5.2 ± 0.3%) compared to the control group (4.2 ± 0.2%), indicating a potential protective effect against apoptosis. The “CTL+OEA-DS” group showed similar data (5.1 ± 0.2%). However, OEA-DS addition to animals with DIO significantly increased the proportion of Bcl-2-positive cells, resulting in levels 88% higher compared to controls (7.9 ± 0.3%) (Figure 4A). Meanwhile, two-way ANOVA revealed significant effects of obesity (F (1, 73) = 63.46; *p* < 0.0001), OEA-DS administration (F (1, 73) = 55.92; *p* < 0.0001), and factor interaction (F (1, 73) = 14.79; *p* = 0.0003) (Figure 4C). Thus, we can conclude that OEA-DS administration inhibits the development of dietary obesity-induced pro-apoptotic responses in the liver and simultaneously stimulates the expression of anti-apoptotic markers.

Western blotting results of liver tissues showed similar dynamics in the changes in both pro- and anti-apoptotic markers. When OEA-DS was administered to obese animals, a significant increase in Bcl-2 expression was observed in the liver (Figure 4E). In the case of the Bax factor, multiple comparison analysis showed no significant differences between groups, but the “DIO” group showed similar dynamics of factor increase, and OEA-DS administration to obese animals decreased it. Two-way ANOVA showed significance of the effects of OEA-DS administration on both Bax (F (1, 8) = 6.248; *p* = 0.0370) and Bad (F (1, 8) = 5.558; *p* = 0.0461), and Bcl-2 (F (1, 8) = 5.579; *p* = 0.0458). Significance of obesity effects was found only for the marker Bax (F (1, 8) = 29.58; *p* = 0.0006), and they did not find significance of the factor interaction for any of the markers.

In general, PCR analysis of liver tissues also demonstrated similar results. DIO development also stimulated the expression of the genes of pro-apoptotic factors Bax and Bad, while OEA-DS administration to obese animals inhibits the expression (Figure 4D). The bcl-2 gene exhibited a different pattern, with the greatest increase in expression observed in the “DIO” group, while OEA-DS administration to obese animals decreased it. Two-way ANOVA for the Bad gene revealed the significance of the effects of obesity (F (1, 20) = 26.72; *p* < 0.0001), OEA-DS administration (F (1, 20) = 7.439; *p* = 0.0130), and their interaction (F (1, 20) = 15.40; *p* = 0.0008). For the bcl-2 gene, only the OEA-DS administration factor was found to be significant (F (1, 20) = 6.546; *p* = 0.0187). For the Bax gene, researchers found the significance of obesity (F (1, 20) = 7.686; *p* = 0.0118) as well as factor interaction (F (1, 20) = 6.124; *p* = 0.0224).

### 3.5. OEA-DS Affects Indices of Lipid Metabolism in the Liver

**Figure 5:**
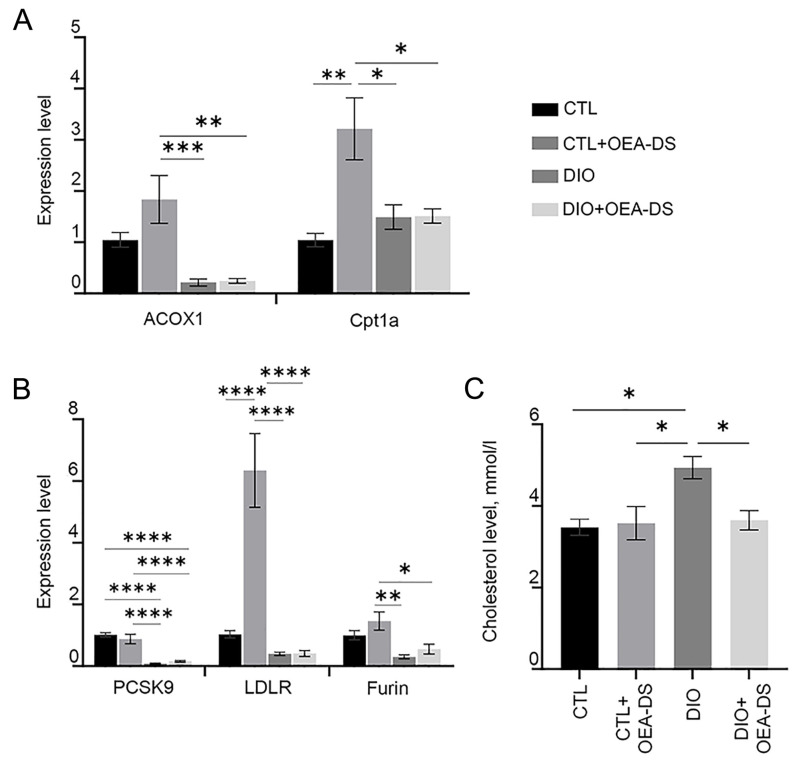
PCR analysis of expression levels of acox1 and cpt1a factors (**A**), and pcsk9, ldlr, and furin factors (**B**). Assessment of cholesterol levels (mmol/L) in serum of animals (**C**). Data are presented as mean ± SEM, * *p* < 0.05, ** *p* < 0.01, *** *p* < 0.001, **** *p* < 0.0001 (Two-way ANOVA followed by Tukey’s multiple comparisons analysis). The number of animals in the group = 3 (for PCR); the period of OEA-DS administration was 2 months; the dose of OEA-DS was 200 mg/kg per day.

Obesity resulted in a 40% increase in liver cholesterol (4.94 ± 0.3 mmol/L) compared to controls (3.48 ± 0.1 mmol/L) and the “CTL+OEA-DS” group (3.58 ± 0.4 mmol/L). OEA-DS therapy in obese animals resulted in normalization of cholesterol levels in obese animals, reducing them by 35% compared to the “DIO” group (3.65 ± 0.2 mmol/L) (Figure 5C). Two-way analysis showed significant effects of obesity (F (1, 17) = 7.142; *p* = 0.0161), and factor interaction (F (1, 17) = 5.895; *p* = 0.0266). Thus, OEA-DS administration may normalize the cholesterol levels in obesity, while having no effect on this parameter with a standard diet (Figure 5C).

The results of the Rt-PCR analysis demonstrate a significant increase in the expression of acox1, cpt1a, ldlr, and furin genes when OEA-DS was administered to animals on a standard diet. At the same time, the development of fatty liver dystrophy leads to inhibited expression of acox1, furin, and ldlr regardless of OEA-DS therapy. For the cpt1a gene, a slight increase in expression was observed as obesity developed, in both in the “DIO” and “DIO+OEA-DS” groups (Figure 5A). In the case of pcsk9, administration of OEA-DS to animals without obesity resulted in a slight decrease the expression of this gene, while diet-induced obesity resulted in a nearly tenfold decrease regardless of OEA-DS administration (Figure 5B). Two-way ANOVA revealed, in the case of the ldlr gene, the significance of obesity (F (1, 20) = 29.86; *p* < 0.0001), OEA-DS administration (F (1, 20) = 19.60; *p* = 0.0003), and factorial interaction (F (1, 20) = 19.48; *p* = 0.0003)) for the ldlr gene. However, in the case of the acox1, pcsk9, and furin genes, a significant effect of the obesity factor alone was detected (found for the genes acox1 (F (1, 20) = 24.00; *p* < 0.0001; for), pcsk9 (F (1, 20) = 94.77; *p* < 0.0001; for), and furin (F (1, 20) = 17.88; *p* = 0.0004). In the case of cpt1b, two-way ANOVA showed the significance of the OEA-DS insertion factors (F (1, 20) = 10.44; *p* = 0.0042), as well as factorial interactions (F (1, 20) = 10.06; *p* = 0.0048).

## 4. Discussion

The development of obesity-related pathologies is driven by key biochemical mechanisms, including the activation of pro-inflammatory signaling pathways [22] and the stimulation of immune system cells [23]. One of the leading mechanisms of obesity-induced MASLD is, firstly, the production of pro-inflammatory factors by adipose tissue itself [24], and secondly, oxidative stress, developing against the background of chronic low-grade inflammation, which also has a damaging effect on hepatocytes [25].

The ability of PPAR-α agonists to suppress inflammation is well established. This effect is mainly mediated through negative regulation of pro-inflammatory transcription factors such as nuclear factor-κB (NF-κB) and activator protein-1 (AP-1) [26], including in the liver [27]. Similar effects have been repeatedly demonstrated in studies for OEA [28] as well as in our previous study for OEA-DS [21]. The present work revealed the ability of OEA-DS to suppress the expression of pro-inflammatory factors in the liver. Also, OEA has been shown in human and animal studies to suppress oxidative stress while activating the antioxidant defense system in the liver, primarily through interaction with PPAR-α [29]. This hypothesis is supported in this study by assessing MDA levels in the liver and in cell culture. Therefore, we can assume that the ability of OEA-DS to suppress inflammation and oxidative stress is mainly mediated by interaction with PPAR-α.

Excessive production of pro-inflammatory signaling factors in the liver tends to promote Kupffer cell activation [30] and their polarization toward a pro-inflammatory M1 phenotype [23], which is accompanied by a secondary increase in the production of pro-inflammatory factors and reactive oxygen species (ROS), contributing to a vicious cycle of inflammation and liver cell damage [31]. In our study, OEA-DS, by suppressing the production of pro-inflammatory cytokines, inhibited inflammation-induced activation of Kupffer cells, as confirmed by immunohistochemical examination. The shift in the proportional distribution of macrophages from CD68-positive to CD163-positive may be due to their polarization toward the anti-inflammatory phenotype M2, which is also characteristic of PPAR-α agonists [32,33]. Similar effects have been previously demonstrated in studies for OEA [34]. Thus, polarization of resident liver macrophages toward the M2 phenotype is an additional aspect of the anti-inflammatory action of OEA-DS.

Activation of hepatocyte apoptosis in the development of fatty liver dystrophy is usually caused by the development of persistent inflammation with subsequent stimulation of Kupffer cells, combined with lipotoxicity and higher production of free radicals. Application of OEA-DS was accompanied by inhibition of liver cell apoptosis in obesity, which is confirmed by both morphological examination—reduction of apoptotic foci in the “DIO+OEA-DS” group compared to the “DIO” group, and immunohistochemical methods—a decrease in the level of pro-apoptotic factors Bad and Bax in the liver is noted against the background of an increase in the level of the anti-apoptotic factor Bcl-2. In this case, the anti-apoptotic effects of OEA-DS are mediated, firstly, by suppression of inflammatory pathways in the liver due to PPAR-α activation, reduction of general oxidative stress, as well as reduction of Kupffer cells activation by pro-inflammatory type and their polarization toward anti-inflammatory phenotype M2. Second, the increased expression of adiponectin receptors in the liver and serum observed in our study may indicate adiponectin-mediated suppression of apoptosis. In animal models and cell cultures, adiponectin suppressed apoptosis through interaction with the PPAR-α receptor [35]. Treatment of animals with adiponectin was also accompanied by a depression in pro-apoptotic markers, including the marker Bax, with a simultaneous rise in the levels of the anti-apoptotic protein Bcl-2 [36]. Therefore, in this case, we can speak about OEA-DS-induced upregulation of adiponectin production, which also contributes to suppression of hepatocyte apoptosis. The lower levels of Bcl-2 protein observed in the “DIO” group, demonstrated by immunohistochemical and Western blot analysis, compared to the high expression of its RNA in the liver, may indicate activation of post-transcriptional and post-translational mechanisms regulating the levels of this factor [37]. In particular, proteins capable of inhibiting Bcl-2 at the protein level may be activated by oxidative stress [38], which typically occurs during the development of fatty liver dystrophy. The presence of increased oxidative stress in the “DIO” group, in turn, is confirmed by the increased MDA levels in liver tissues.

In relation to lipid metabolism regulation, the main effects of PPAR-α agonists are the activation of fatty acid beta-oxidation, inhibition of de novo lipogenesis [39], and a decrease in the processes of triacylglyceride synthesis in the liver [40]. These effects, previously demonstrated for OEA [41], are confirmed in the present study for OEA-DS directly by PCR analysis of liver beta-oxidation enzymes and indirectly by liver weight assessment, which may partly explain the inhibition of weight gain in mice in our previous study [21]. Activation of adiponectin expression may also contribute to the enhancement of lipolytic processes in the liver, since adiponectin has been shown to be active in initiating beta-oxidation and simultaneously inhibiting de novo lipogenesis [42]. At the same time, PPAR-α agonists increased adiponectin receptor expression [43]. Accordingly, OEA-DS, through activation of PPAR-α, as well as through the effect on adiponectin expression, causes an increase in beta oxidation of fatty acids, slowing down lipid accumulation in the liver, and, thus, inhibiting the development of fatty liver dystrophy.

The serum cholesterol level reduction in obese animals treated by OEA-DS is probably also due to interaction with PPAR-α. PPAR-α-dependent cholesterol lowering, mediated by downregulation of the PCSK9 activity, a natural inhibitor of the low-density lipoprotein receptor (LDLr), has also been identified for the widely used hypocholesterolemic drug fibrates [44,45]. This class of drugs is characterized by a dual mechanism of action: binding to the appropriate sites on the PCSK9 gene promoter and suppression of PCSK9 gene transcription [44]; and at the post-translational level—through activation of PC5/6A and furin, proteins that perform proteolytic cleavage of PCSK9 protein [46].

Thus, taking into account the effect on serum cholesterol levels and the obtained data from PCR analysis of liver tissue, it can be assumed that OEA-DS has effects similar to fi-brates, which is also confirmed by an increase in the level of LDL expression in animals with a standard diet that received dietary supplements, compared with the control group. There is also a trend toward increased furin and decreased pcsk9 in non-obese groups. At the same time, the absence of similar dynamics in the case of groups with dietary obesity can be associated with the lesion of functional liver cells and a decrease in the production of relevant proteins. And probably, the anti-apoptotic effect of OEA-DS indirectly affects the enhancement of pcsk9 expression in the “DIO+OEA-DS” group. Also, high serum cholesterol levels in the “DIO” group against the background of reduced pcsk9 expression can be explained by excessive intake of exogenous cholesterol in the diet. Nevertheless, further studies of pcsk9 and ldlr protein levels in the liver, as well as evaluation of PC5/6 levels during OEA-DS therapy, are required to definitively confirm the presence of this “dual mechanism” in OEA-DS. In addition to the above-mentioned effects, the contribution of adiponectin to the hypocholesterolemic effects of OEA-DS cannot be excluded. OEA has been experimentally demonstrated to inhibit monocyte adhesion to endothelial cells by reducing the amount of endothelial adhesion molecules induced by TNFα, as well as inhibiting the conversion of macrophages into foam cells and preventing endothelial cell activation [47]. Also, adiponectin caused a decrease in CRP levels and increased nitric oxide (NO) production by endothelial cells [48]. Moreover, it inhibited cell proliferation and ROS production induced by LDL oxidase during atherosclerotic plaque formation [49]. It is known that adiponectin deficiency is accompanied by a decrease in NO in vascular walls and promotes leukocyte adhesion activation, causing chronic vascular inflammation [50]. The OEA-DS-mediated upregulation of PPAR-γ receptor expression, observed both in liver tissues during the development of dietary obesity and in cell culture, indicates that OEA-DS may act as a PPAR-γ agonist, which was also previously demonstrated experimentally [51]. Regarding metabolic effects due to PPAR-γ activation, although some studies demonstrate an association between its increased expression and the development of fatty liver dystrophy [52], there are also examples of studies where PPAR-γ activation led to reduced inflammation in MASLD [53,54]. In animals lacking PPAR-γ, macrophage polarization toward the M2 phenotype was impaired, while PPAR-γ activation was accompanied by an inhibition of the M1 macrophage polarization [55]. Finally, agonism with PPAR-γ resulted in low levels of TNFα and IL6 [56] and adiponectin [57,58,59] in adipose tissue. The downregulation of PPAR-γ expression in the liver observed in our study during the development of diet-induced obesity may be due to significant damage to liver cells, and the normalization of its level during OEA-DS therapy may be the result of a decrease in the intensity of inflammatory processes in the liver and apoptosis.

In the present study, none of the side effects characteristic of OEA and previously described in experimental studies were observed in animals [60]. This may be due to the higher doses of OEA (300 and 600 mg/day) used in the aforementioned study compared to the lower doses used in the present study.

For the combined formation of dietary obesity, hypercholesterolemia, and MASLD simultaneously, a high-fat, high-cholesterol diet is probably the most favorable model due to the following components. High fat promotes weight gain and fat deposition, a key feature of obesity; adding cholesterol to the diet directly increases blood cholesterol levels, modeling hypercholesterolemia; and the combination of these factors leads to fat accumulation in the liver, the development of steatosis, and ultimately MASLD and steatohepatitis [61,62]. Diets high in fructose or sucrose can also cause obesity, especially if consumed over a long period of time. However, they are less effective in raising cholesterol levels than high-fat, high-cholesterol diets. They mainly affect triglyceride and LDL levels. These types of diets can also cause MASLD, but the mechanism is different. Fructose mainly promotes de novo lipogenesis in the liver rather than the accumulation of exogenous cholesterol [63]. Thus, a high-fat diet combined with high cholesterol intake is the most optimal option for the development of the combined pathology of dietary obesity, hypercholesterolemia, and NASH. The length of the trial also makes logical sense. In general, 2 months is often considered a sufficient time frame for the development of diet-induced obesity in mice, especially when a high-fat diet is used. However, the rate of development of obesity and the severity of metabolic abnormalities may depend on several factors, such as the specific strain and the age of the animals. The C57BL/6 strain used in the study is more susceptible to obesity than others [64]. Also, young mice may be more susceptible to obesity than older mice [65].

However, this work clearly has a number of limitations. Obesity was induced by a diet high in fat and cholesterol, and did not take into account genetic factors, lifestyle, and other comorbidities associated with obesity in humans. Although this study suggests that OEA-DS acts through PPAR-α, the mechanism of action may be more complex and involve other pathways, such as metabolic effects of OEA-DS mediated by activation of the GPR119 receptor [66]. It is also worth mentioning that the study only evaluated certain markers of inflammation, oxidative stress homeostasis, and apoptosis; however, this study did not evaluate the effects of OEA-DS on insulin resistance, a major pathophysiological factor in the development of MASLD [67]. The choice of sex of the animals in the study can significantly influence the results. Using females allows the effect of estrogen on metabolism to be taken into account [68], which can be important when studying obesity [69] and related diseases [70], and has a number of additional benefits, including a lower propensity to become obese [71], less variability in data [72], and greater social tolerance [73]. However, results obtained in females may not be fully generalizable to males and vice versa. Therefore, it is desirable to conduct studies in both males and females to get a more complete picture. To assess protein expression by Western blotting, loading control (α-tubulin) was performed on a separate gel. Although this approach is generally accepted, it does not completely eliminate the possibility of systematic errors due to differences in the amount of loaded protein between samples. To better demonstrate the observed differences in protein expression, alternative loading control methods, such as Ponceau staining or total protein normalization, are planned to be used in future studies to improve the accuracy and reliability of Western blotting results. Finally, it is important to clarify that other types of nuclear receptors that function as transcription factors also play important roles in maintaining lipid homeostasis and controlling inflammation in the liver. The farnesoid X receptor (FXR) mainly protects the liver from excessive fat accumulation and inflammation [74], while the liver X receptor (LXR) regulates cholesterol metabolism and can have both positive and negative effects on inflammation depending on the specific conditions [75]. An imbalance in the activity of these receptors may also contribute to the development of MASLD and other liver diseases. Therefore, additional studies are needed to better understand the multifactorial protective effects of OEA-DS in coexisting metabolic pathologies.

## 5. Conclusions

PPARs are currently one of the most promising options for the comprehensive treatment of MASLD and are attracting increasing research attention. In pathologies such as lipid and glucose metabolism disorders, inflammation, obesity, decreased insulin resistance, and atherosclerosis, which may lead to secondary liver damage, the potential benefits of PPAR agonists are well recognized. The efficacy of PPARs as a target for the treatment and prevention of MASLD is evident due to their functional position at the intersection of lipid metabolism, energy balance, and inflammation. OEA-DS, being an agonist of the aforementioned receptors, may be involved in various pathophysiological pathways in the MASLD development, reducing liver damage and slowing disease progression. Adiponectin, affecting multiple pathogenetic links of MASLD, is a very promising pharmacological target. Thus, OEA-DS, modulating the activity of adiponectin, in combination with its expressed anti-inflammatory effect, may be particularly relevant in the complex therapy of MASLD, especially if this pathology is associated with such metabolic complications as obesity and hypercholesterolemia.

## Data Availability

The original contributions presented in this study are included in the article/Supplementary Materials. The datasets generated during the current study are available from the corresponding author on reasonable request.

## Supplementary Materials

The following supporting information can be downloaded at: https://www.mdpi.com/article/10.3390/pathophysiology32020016/s1, Images of the Western blot bands are provided in Supplementary Materials S1; full images of the gels are provided in Supplementary Materials S2.
