# Supplementary material for: Hepatoprotective and Antiatherosclerotic Effects of Oleoylethanolamide-Based Dietary Supplement in Dietary-Induced Obesity in Mice
Source: Pathophysiology. 2025 Apr 18;32(2):16. doi: 10.3390/pathophysiology32020016 (PMC12015875; doi:10.3390/pathophysiology32020016)
Supplement: Supplementary file 1 [file pathophysiology-32-00016-s001.zip › Supplementary materials S1.pdf]

A - Western blot analysis of serum, graphs for these bands are shown in Fig. 2D.

B - Western blot analysis of liver tissue, graphs for these bands are presented in Fig. 2D, 2E, 3G, 4E.

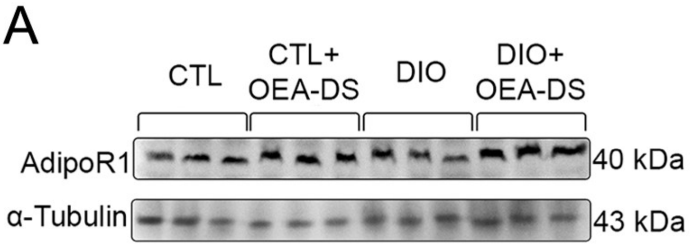

The following steps were taken in the execution of the western blotting with loading control on a separate gel (single-marker analysis):

1. Sample preparation:
An equivalent amount of protein was added to all wells of each gel.
2. Protein separation:
The prepared samples were separated by electrophoresis in a polyacrylamide gel (SDS-PAGE). Concurrently, a separate gel was prepared in which the same samples were added in equivalent amounts. This gel was used to evaluate the loading control.
3. Transfer to membrane:
Proteins from the main gels and control gel were transferred to membranes (PVDF).
4. Blocking and incubation with antibodies:
Each membrane was blocked to prevent nonspecific antibody binding and then incubated with primary antibodies to only one target marker, while the control membrane was incubated with antibodies to tubulin.
5. Incubation with secondary antibodies:
All membranes were incubated with appropriate horseradish peroxidase-conjugated secondary antibodies
6. Detection:All membranes were detected only once by chemiluminescence for the respective markers.
7. Analysis:The intensity of bands on the primary membrane corresponding to the target marker was normalized to the intensity of loading control bands on the individual membrane.

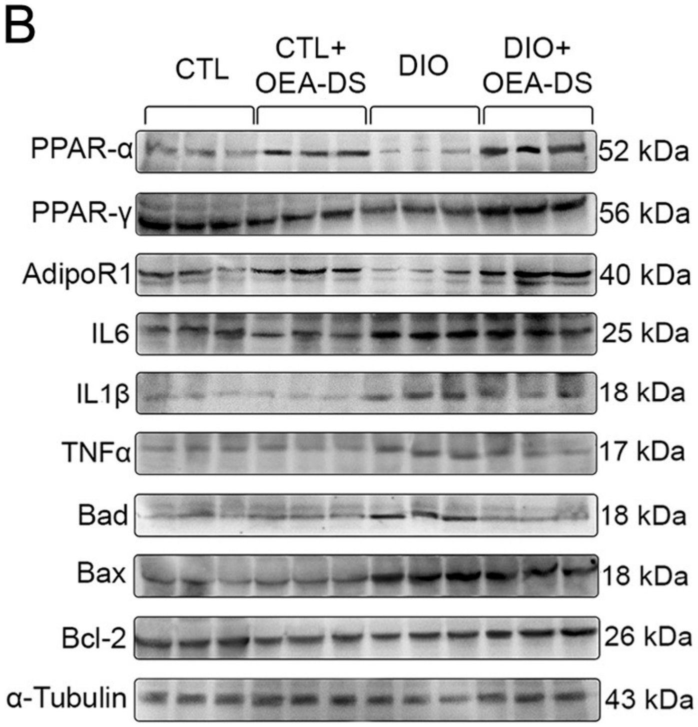

The rationale for employing this technique in our particular case is as follows:

1.The loading control is executed on a distinct gel, thereby circumventing potential complications associated with the concurrent detection of multiple proteins on a singular membrane (e.g., overlapping bands—in our case, numerous markers possess close molecular masses—to tubulin and to each other; nonspecific antibody binding).
2.The omission of antibody washing and membrane reuse steps can enhance the sensitivity of target protein detection.
3.The omission of antibody washout and re-incubation steps mitigates the risk of errors and artifacts.
