# Supplementary figures and images for: Hepatoprotective and Antiatherosclerotic Effects of Oleoylethanolamide-Based Dietary Supplement in Dietary-Induced Obesity in Mice
Source: Pathophysiology. 2025 Apr 18;32(2):16. doi: 10.3390/pathophysiology32020016 (PMC12015875; doi:10.3390/pathophysiology32020016)

**Figure S2.**

1)Serum

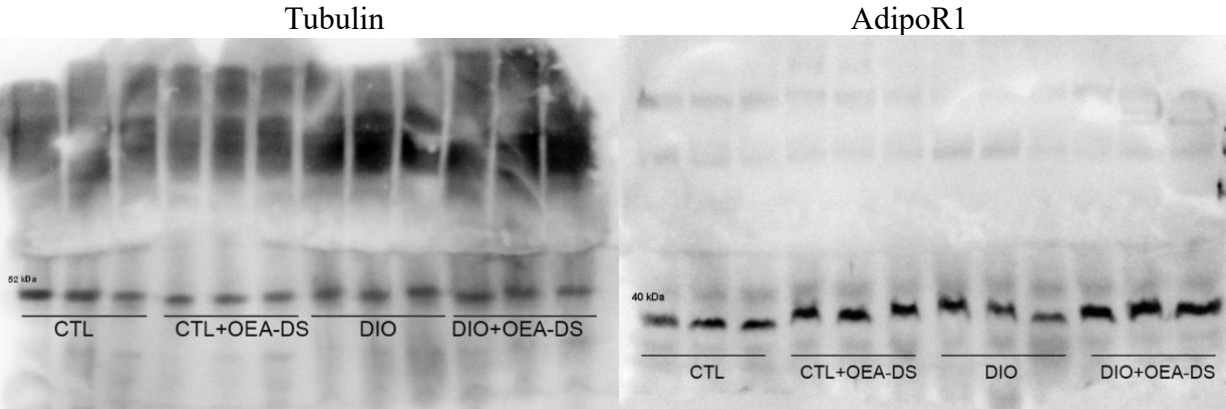

2)Liver

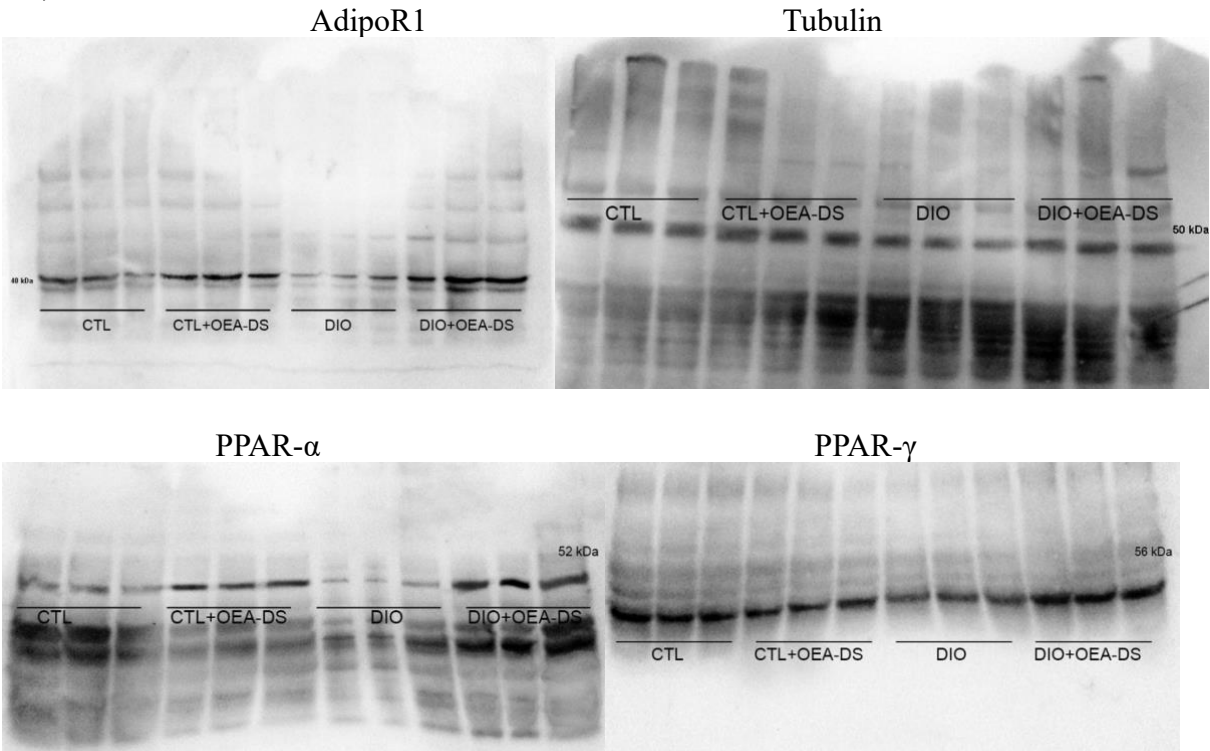

3)HepG2

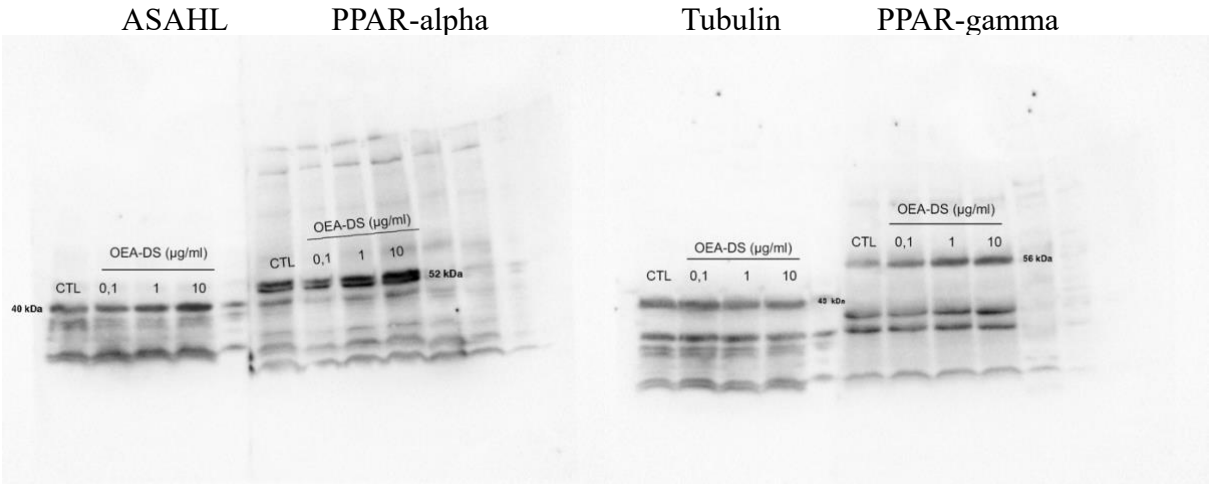

**Figure S3.**

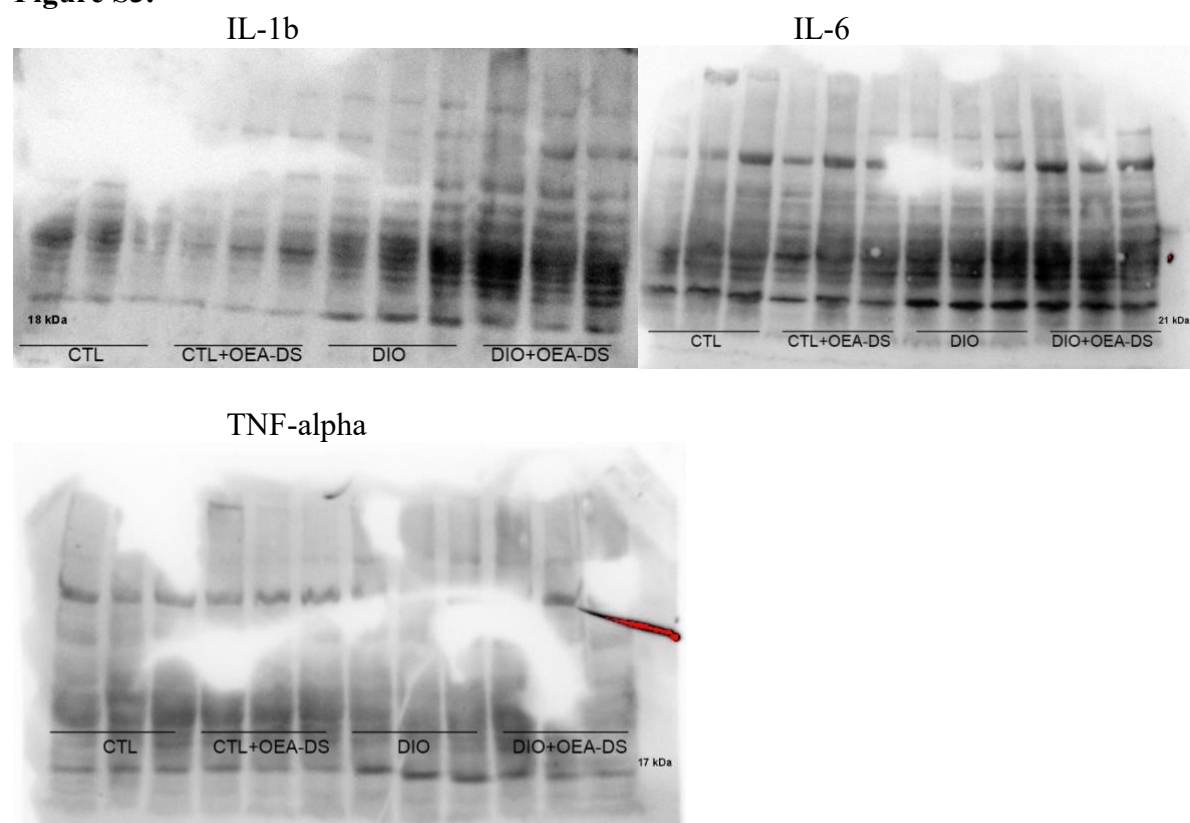

**Figure S4.**

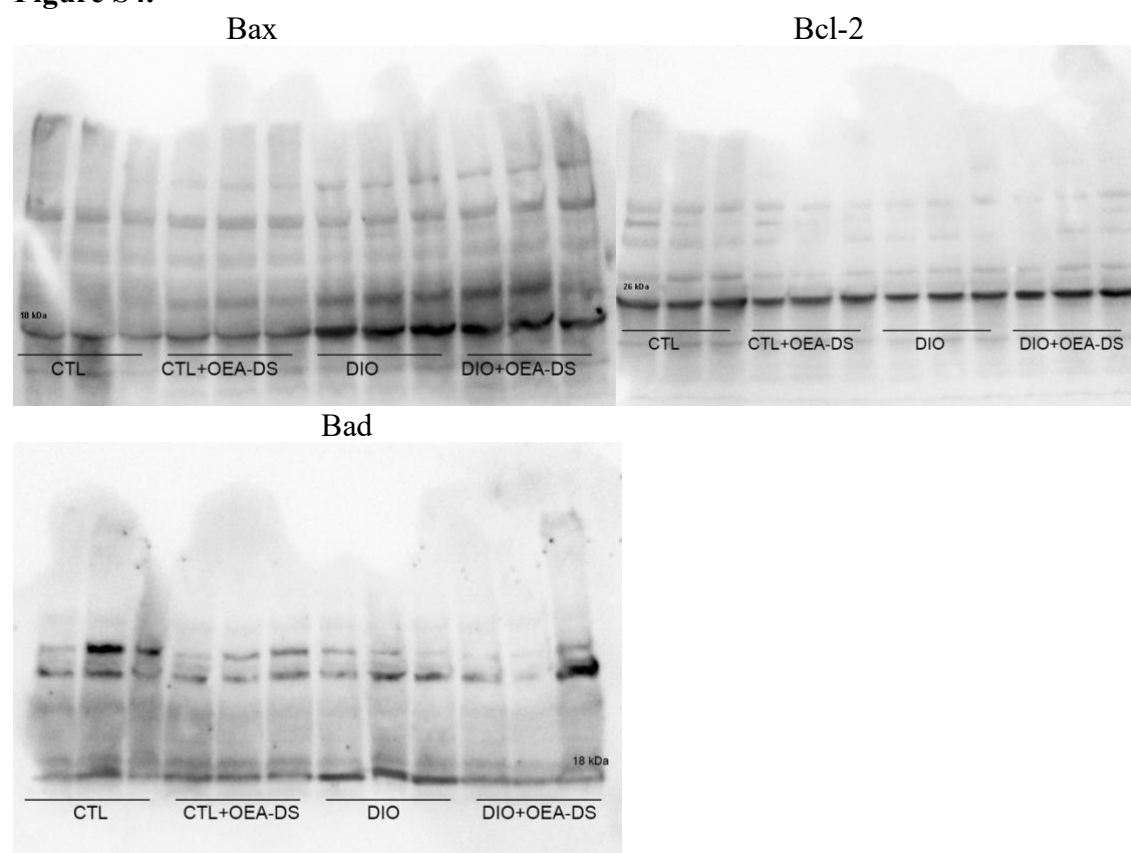

Supplement: Supplementary file 1 [file pathophysiology-32-00016-s001.zip › Supplementary materials S2.pdf]
